# Patients’ and Clinicians’ Experiences Using a Real-Time Remote Monitoring System for Chemotherapy Symptom Management (ASyMS): Qualitative Study

**DOI:** 10.2196/53834

**Published:** 2024-12-03

**Authors:** Lisa McCann, Liane Lewis, Olubukola Oduntan, Jenny Harris, Andrew Darley, Geir V Berg, Simone Lubowitzki, Katy Cheevers, Morven Miller, Jo Armes, Emma Ream, Patricia Fox, Eileen Patricia Furlong, Alexander Gaiger, Grigorios Kotronoulas, Elisabeth Patiraki, Stylianos Katsaragakis, Paul McCrone, Christine Miaskowski, Antonella Cardone, Dawn Orr, Adrian Flowerday, Simon Skene, Margaret Moore, Nicosha De Souza, Peter Donnan, Roma Maguire

**Affiliations:** 1 Digital Health and Wellness Group (DHaWG) Department of Computer and Information Sciences University of Strathclyde Glasgow United Kingdom; 2 University Hospital Jena Jena Germany; 3 University of Surrey School of Health Sciences Guildford United Kingdom; 4 School of Nursing, Midwifery and Health Systems University College Dublin Dublin Ireland; 5 Section for Nursing Faculty of Social and Health Sciences Inland Norway University of Applied Sciences Elverum Norway; 6 Department of Internal Medicine Division of Hematology and Hemostaseology Medical University of Vienna Vienna Austria; 7 Hastings and Rother Healthcare Hastings United Kingdom; 8 School of Medicine, Dentistry & Nursing University of Glasgow Glasgow United Kingdom; 9 School of Health Sciences National and Kapodistrian University of Athens Athens Greece; 10 Institute for Lifecourse Development Faculty of Education, Health & Human Sciences University of Greenwich London United Kingdom; 11 Department of Anesthesia and Perioperative Care University of California San Francisco, CA United States; 12 Department of Physiological Nursing University of California San Francisco, CA United States; 13 Cancer Patients Europe Brussels Belgium; 14 NHS 24 Glasgow United Kingdom; 15 Docobo Limited Leatherhead United Kingdom; 16 Surrey Clinical Trials Unit University of Surrey Guildford United Kingdom; 17 Biomathematics and Statistics Scotland (BioSS) The Rowett Institute Aberdeen United Kingdom; 18 Division of Population Health and Genomics School of Medicine University of Dundee Dundee United Kingdom

**Keywords:** cancer, clinician experiences, digital interventions, patient experiences, remote monitoring, qualitative methods

## Abstract

**Background:**

Patients receiving chemotherapy require ongoing symptom monitoring and management to optimize their outcomes. In recent years, digital remote monitoring interventions have emerged to provide enhanced cancer care delivery experiences to patients and clinicians. However, patient and clinician experiential evaluations of these technologies are rare. Therefore, we explored user experiences and perceptions of one such intervention—Advanced Symptom Management System (ASyMS)—after its scaled deployment in the context of the Electronic Symptom Management System Remote Technology (eSMART) trial. The eSMART trial was a large, multicenter randomized controlled trial to evaluate the efficacy of ASyMS in 12 clinical sites in 5 European countries.

**Objective:**

In this qualitative study, both patients’ and clinicians’ experiences of using ASyMS for up to 6 cycles of chemotherapy were explored to understand the impact of ASyMS on patients’ experiences, clinical practice, and supportive care delivery.

**Methods:**

For this analysis, individual, semistructured, one-to-one interviews with 29 patients with breast, colorectal, and hematological cancers and 18 clinicians from Austria, Greece, Ireland, Norway, and the United Kingdom were conducted. Interviews focused on patients’ and clinicians’ experiences of using ASyMS, care organization and changes in practice following the introduction of ASyMS, perceived changes in care associated with the use of ASyMS, and its potential for future integration into routine chemotherapy care pathways.

**Results:**

Thematic analysis identified several themes that describe patients’ and clinicians’ experiences using ASyMS. One central orienting theme—ASyMS as a facilitator of change—was supported by 5 key themes associated with human and technology monitoring: reassurance, enhanced communications and relationships, knowing what is “normal” and what is to be expected, enhancing cancer care experiences, and informing future cancer care.

**Conclusions:**

This study is the first to evaluate both patients’ and clinicians’ experiences of using a digital health intervention to remotely monitor chemotherapy symptoms across 5 countries. Experiences with ASyMS were positive from both patients’ and clinicians’ perspectives, although some improvements to support the wider-scale rollout and sustained implementation were identified. Overall, this study demonstrates that real-time remote monitoring systems can help patients feel more reassured during their chemotherapy treatments and can help clinicians provide the right care, at the right time, and in the right place.

**Trial Registration:**

ClinicalTrials.gov NCT02356081; https://clinicaltrials.gov/study/NCT02356081

**International Registered Report Identifier (IRRID):**

RR2-10.1136/bmjopen-2016-015016

## Introduction

### Background

Patients receiving chemotherapy experience a range of adverse effects. Several of these effects can be severe or life-threatening, particularly if not reported promptly and managed effectively [1]. Traditionally, chemotherapy symptom management relies on patient recall, but this approach is limited because it is prone to recall bias and inaccuracy [2]. The lack of real-time, accurate reporting of symptoms impedes timely interventions [3]. Therefore, a growing momentum exists within cancer care services to introduce electronic patient-reported outcome measures (PROMs) to better support real-time clinical practice [4]. Remote patient monitoring systems that facilitate regular assessment of symptoms [5] can influence changes to clinical practice by assisting rapid clinical decision-making and interventions and supporting improvements in patients’ experiences of cancer care [6] and outcomes [7].

Digital interventions for symptom monitoring in cancer care are critical due to the increasing global burden of cancer [8], the increasing complexity of cancer treatments [9], and the shortage of cancer care health professionals worldwide [10]. Implementing innovative models and services such as remote monitoring technologies can influence positive changes to deliver safe and optimal cancer care [6,11,12].

Therefore, learning from and reflecting on the experiences of users is key to understanding the potential for sustainable real-world implementation of digitally enabled systems into clinical care. To support changes in clinical practice associated with new digitally driven models of cancer care, we need an increased understanding of patients’ and clinicians’ experiences of using such digital remote patient monitoring systems. We also need to consider how these systems can be implemented into different health care systems. Thus, in this study conducted across 5 European countries, we evaluated the use of Advanced Symptom Management System (ASyMS) within a large-scale randomized controlled trial (RCT) called Electronic Symptom Management System Remote Technology (eSMART) [13] and explored patients’ and clinicians’ experiences and perceptions of using ASyMS.

### The Digital Intervention and Deployment Context: ASyMS and the eSMART Study

#### The Digital Intervention

ASyMS is a mobile phone–based, real-time, remote patient monitoring system used to assess and manage chemotherapy-related toxicities experienced by adult patients with cancer. It is a stand-alone, nurse-led, purpose-built, 24-hour anticipatory care system with integrated evidence-based clinical algorithms and alerts that provide rapid access to specialist staff in the patient’s cancer hospital and timely initiation of appropriate interventions [13-15]. In eSMART, patients were provided with a mobile phone with the ASyMS app installed to complete an app-based electronic Daily Chemotherapy Toxicity Assessment Questionnaire (DCTAQ) [16]. The DCTAQ assesses 10 common chemotherapy-related symptoms (nausea, vomiting, diarrhea, constipation, hand-foot syndrome, mucositis, paresthesia, flu-like symptoms, fatigue, and pain). An opportunity exists to enter a free-text report for up to 6 additional symptoms [13-16]. In eSMART, patients used ASyMS for a maximum of 6 cycles of chemotherapy [15].

The integrated clinical risk algorithms and embedded alerts within ASyMS are informed by the Common Terminology Criteria for Adverse Events (CTCAE V4.0) [17]. It uses a green, amber, and red traffic light system to indicate the severity, distress, and bother of symptoms reported, respectively. Alerts are generated in real time from the patient’s device to a clinician’s device at the local cancer center through a secure server. Clinicians access patient reports via a dedicated website. This approach facilitates time-bound clinician responses according to the alert level (amber alerts=8 hours and red alerts=30 minutes). Additional details on ASyMS and the RCT are described elsewhere [13-15].

All participants received training from researchers or clinicians at each clinical site to use ASyMS before commencing in the RCT. Manuals and videos were also provided for all participants for any ongoing training needs during the study.

#### Deployment Context

We conducted an RCT to evaluate the efficacy of ASyMS in 12 clinical sites in 5 European countries with public and private health care systems: Austria, Greece, Ireland, Norway, and the United Kingdom [13-15]. Patients with breast, colorectal, or hematological cancers were included in this RCT. Fuller details on patient inclusion criteria, exclusion criteria, and characteristics for the overall RCT are published elsewhere [15]. The findings from the RCT indicate that ASyMS use led to significant and sustained reductions in patients’ symptom burden during chemotherapy as well as significant improvements in anxiety, quality of life, self-efficacy, and reductions in unmet supportive care needs [15].

### Study Aims

The aim of the study is to explore patients’ and clinicians’ experiences of using ASyMS during chemotherapy and understand its impact on patients’ experiences, clinical practice, and supportive care delivery.

## Methods

### Study Setting

Participants were recruited from cancer centers in the 5 partner countries: Austria, Greece, Ireland, Norway, and the United Kingdom. Patient interview participants were sampled purposively from the intervention population from the 3 diagnostic groups and were recruited either during their intervention use or within 1 year of completing the RCT. Clinicians were approached directly by members of the local country research teams and were sampled purposively to participate based on their knowledge, exposure, and use of ASyMS in the eSMART study.

### Participant Recruitment

Patients randomized to the intervention were sampled purposively across the 3 cancer diagnoses, 5 countries, and at various times (during, at completion, or within 1 year of completion of chemotherapy). Patients were approached by telephone or face-to-face and invited to participate by members of the research team. Of the 415 patients assigned to the RCT intervention, 29 were interviewed. Across the 5 countries, clinicians who used ASyMS were approached by email or face-to-face by members of the research team and invited to participate in interviews. In total, 18 consented to participate. All interviews were conducted face-to-face or by telephone between September 2018 and January 2019.

### Procedures

In total, 9 members of the in-country research team at the clinical site (7 female and 2 male members) with academic or nursing backgrounds and with native language skills conducted one-to-one patient and clinician interviews. Semistructured interviews were conducted in clinic and hospital environments using topic guides developed for the study (Multimedia Appendices 1 and 2). Open-ended questions were designed to explore various broad areas, including patients’ and clinicians’ experiences of using ASyMS, care organization and changes in practice following the introduction of ASyMS, perceived changes in care associated with the use of ASyMS, and its potential for future integration into routine chemotherapy care pathways.

Prior to the interviews, researchers participated in interview training sessions delivered via videoconference in small groups and received training resources before and after these sessions to standardize the interview processes. To ensure quality control of the data collection procedures, LM and LL listened to initial interviews conducted by each interviewer to provide reflective and constructive feedback prior to subsequent interviews. The length of patient and clinician interviews varied; the shortest were 6 and 5 minutes, respectively, and the longest were 28 and 57 minutes, respectively.

Individual face-to-face or telephone interviews were conducted over a 5-month period and were recorded on secure, password-protected, and encrypted devices. Anonymized interview files were stored in a secured cloud-based folder with tiered access managed by LL. All interviews were transcribed verbatim. Interviews conducted in Austrian, Greek, and Norwegian were translated and transcribed simultaneously and then checked by a member of the research team with native language skills. In the event of any errors or inaccuracies, corrections were made to transcribed text prior to the commencement of data analyses.

### Ethical Considerations

The eSMART study was registered on ClinicalTrials.gov (NCT02356081) and was granted ethics approval from the National Health Service Lothian Southeast Scotland Research Ethics Committee 02 (14/SS/1062). The study received National Health Service Research and Development approvals and local clinical site ethics approvals in each partner country prior to data collection commencement. All participants provided written informed consent prior to participating in an interview. All interview transcripts were anonymized prior to analysis; participants are referred to by an allocated ID number only. No compensation of any kind was provided to participants.

### Analysis

Thematic analysis was used to analyze the data [18] and was conducted by a team of researchers (LM, LL, and OO). Our a priori focus was on exploring changes in practice arising from digitally enabled chemotherapy supportive care services. This meant a central orienting theme of “ASyMS as a facilitator of change” was somewhat deductively identified prior to open coding of data to ensure codes and themes identified in the data reflected perceptions, experiences, and meanings reported by participants [18].

A codebook was developed to ensure consistency in analytic approaches, given the team-based approach to analysis. Brief descriptions of each theme and subtheme were described in the codebook as a reference for each member of the analysis team. One team member (LM) assumed responsibility to update, revise, and maintain the codebook to ensure consistency and minimize the risk of any ambiguities in its evolution over time. Team meetings were held on a regular basis throughout the qualitative analyses to ensure consistency in the approach and discuss the emergent data to inform decisions on our reaching data saturation. In addition, intercoder reliability checks mitigated against potential inconsistencies and disparities in data analysis and interpretation across team members.

A coding comparison scheme compared the coding conducted by members of the analysis team. Of the 47 interviews conducted, 20% (n=9) of the transcripts were randomly selected for coding comparison to capture any variations. Transcripts were coded by 2 members of the team, as interviews were completed, translated, and transcribed. In addition, quality assurance within the random sample was ensured with the inclusion of interview transcripts conducted by different persons, different diagnoses, and across different stages of chemotherapy. As part of the intercoder reliability assessments, the team discussed problems with coding definitions and clarified these definitions to improve consistency. The coding framework is available (Multimedia Appendix 3). The qualitative software analysis software NVivo (QSR) was used throughout the analysis processes to organize and manage the data.

## Results

### Patient Characteristics

In total, 29 patients were interviewed across the 5 countries. Patient characteristics are shown in Table 1. Patients’ mean age was 48.1 (SD 14.71; range 19-78) years; two-thirds of patients (n=19, 66%) were diagnosed with breast cancer, and most were female (n=22, 76%).

**Table 1:** Demographic and clinical characteristics of patients participating in an interview.

Characteristics				Patients (n=29)	
**Cancer diagnosis, n (%)**					
	Breast cancer		19 (66)		
	Colorectal cancer		3 (10)		
	Hematological cancer		7 (24)		
**Sex, n (%)**					
	Female		22 (76)		
	Male		7 (24)		
**Age (years)**					
	Mean (SD)		48.1 (14.71)		
	Range		19-78		
**Country, n (%)** ^a^					
	Austria		9 (31)		
	Greece		7 (24)		
	Ireland		5 (17)		
	Norway		3 (10)		
	United Kingdom		5 (17)		
**Timing of participation in the interview**					
	**During RCT** ^b^				
		Patients, n (%)			9 (31)
		Chemotherapy cycles, mean (SD)			4.6 (1.1)
		Chemotherapy cycles, range			3-6
	**After RCT**				
		Patients, n (%)			20 (69)
		Months since RCT completion, mean (SD)			3.9 (2.36)
		Months since RCT completion, range			1-7

^a^Some sites were able to recruit more participants from >1 diagnostic group so recruited more patients.

^b^RCT: randomized controlled trial.

### Clinician Participant Characteristics

In total, 18 clinicians were interviewed across all the 5 countries (Table 2). Most were female (n=13, 72%) and nurses (n=15, 83%) likely due to ASyMS being a nurse-led intervention.

**Table 2:** Demographic characteristics of clinicians participating in an interview.

Characteristics		Clinicians (n=18)
**Sex, n (%)**		
	Female	13 (72)
	Male	5 (28)
**Age (years) (n=11)**		
	Mean (SD)	39.8 (12.62)
	Range	27-60
**Country, n (%)**		
	Austria	2 (11)
	Greece	3 (17)
	Ireland	4 (22)
	Norway	3 (17)
	United Kingdom	6 (33)
**Roles, n (%)**		
	Nurses	15 (83)
	Medical director	1 (6)
	Clinical nurse manager	2 (11)

### Central Orienting Theme: ASyMS as a Facilitator of Change

#### Overview

ASyMS was perceived as a facilitator of change across the different health care settings for either patients, clinicians, or both. Patients highlighted the ease of reporting symptoms, and clinicians reflected on the ease of responding to flagged symptoms, together creating targeted and timely care in what is usually a complex treatment process.

**Figure 1:**
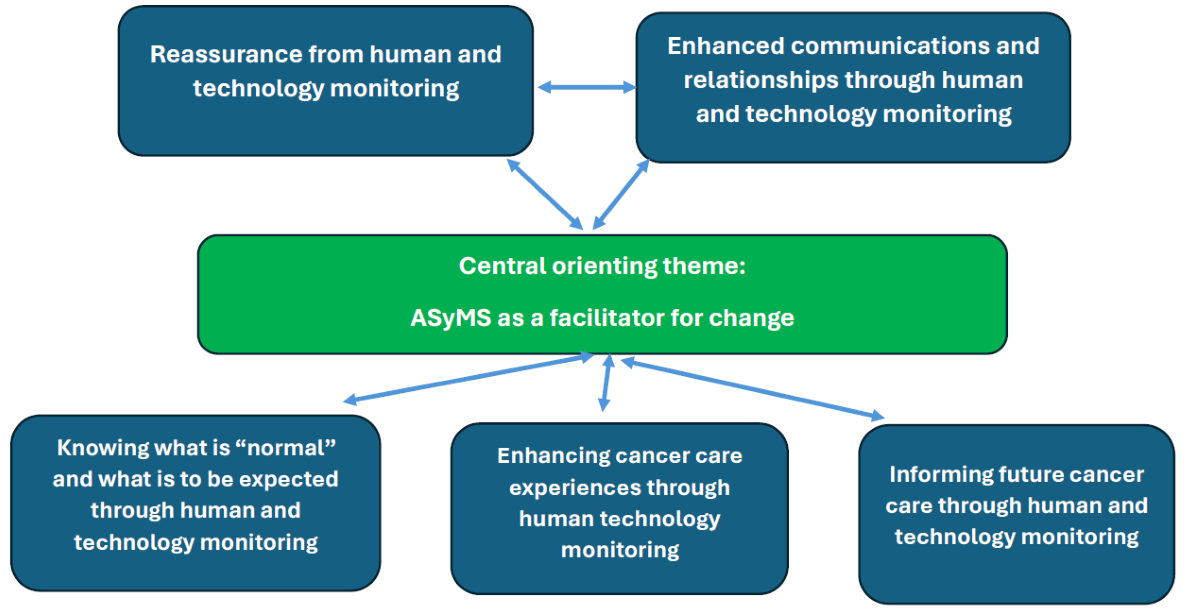
Central orienting theme and supporting themes identified from analysis of patient and clinician interviews. ASyMS: Advanced Symptom Management System.

The combined reassurance, enhanced communications, knowledge, and improved cancer care experiences emerged from ASyMS’ interactions between human and technology monitoring. The patterns identified in the data that increased our understanding of how and why participants saw ASyMS as a facilitator of change are summarized in Figure 1. Key quotations that support these themes are embedded within the text.

#### Theme 1: Reassurance From Human and Technology Monitoring

ASyMS was a mechanism that provided reassurance to patients during their chemotherapy treatments. Patients spoke positively about the human-centric proposition of ASyMS. They commented that while they were using technology in a new way to monitor symptoms, it was real humans, not machines, who reacted and responded to their clinical needs, and they found this reassuring:

What I think was incredibly good, is this real-life support, so that as soon as something [that] is outside of the norms and crosses certain values, that you then get real feedback and are called and can talk to someone, especially during the weekends.

Austria, Patient ID 4

It felt safe, I answered every day, and if anything at all showed up you called and we talked about it. That’s a great reassurance.

Norway, Patient ID 3

And it was an incredible tool in a truly difficult journey and I felt that there was an invisible companion for me during this process and this was very nice.

Greece, Patient ID 2

Clinicians were cognizant of the greater reassurance that this approach afforded patients in the intervention group.

I think that the eSMART system meant that they were feeling a lot safer at home. Because it can be scary to go out of the hospital and you know that there are quite a lot of severe symptoms you can receive. And the patients always told us they were feeling a lot safer at home knowing that they had the device and could answer and then receive help...

Norway, Clinician ID 2

And in regard to their symptoms. For starters, the patient felt a form of security. That I have someone that I can talk to at any time, because they had their cell phone during the entire 24 hours. And they could contact, while they also had an immediate answer to their issue, it helped them a lot, in regard to their specific symptoms.

Greece, Clinician ID 3

However, for some patients, the daily PROM entries became too much as their treatment progressed, and their symptoms stabilized. The constant connection with the clinical setting was inadvertently fatiguing; and therefore, some patient perceptions of the intervention were more negative.

Patient ID 3 [Austria]: In the beginning I tried to do it daily. But at one point it got a bit too exhausting...Near the end I knew how to treat something. And it was exhausting to receive a call, despite not having any problems.

Interviewer: Can you roughly estimate the point in time when it got exhausting?

Patient ID 3: Yes, what was happening then...September, October, November...so after 3-4 months. Roughly. I didn’t need any more help then.

#### Theme 2: Enhanced Communications and Relationships Through Human and Technology Monitoring

Both patients and clinicians frequently referred to ASyMS’ contribution to enhanced dyadic communication and relationships. The technology facilitated clinicians’ knowledge of patients and therefore their provision of more personalized and individualized care. ASyMS was perceived as a relationship enabler by clinicians.

I think the most useful [thing] was getting to know the patients...So when you did go into the system, if by some chance you couldn’t get hold of them there and then, you knew that the patient was, you know, clued up enough to ring you if there was something really wrong.

United Kingdom, Clinician ID 3

Interviewer: Do you do something now that you did not do before?

Clinician ID 1 [Austria]: What I did not do before, regarding...I think that I am simply listening better, and I am trying to ask many questions also, listen and not only give suggestions and advice but really focus on the patient and what they really can do. Because we, as caregivers, tend a lot to give suggestions, suggest A, B, C or D and see what the patient does of that or not. Now I look why they might not be able to do something that I have suggested. So that I try to approach the patient in a more individual way and work towards a solution.

Poignantly, from a patient’s perspective, it was evident that the technology enhanced their relationship with their clinicians.

I felt that I had some people who cared about me.

Greece, Patient ID 3

#### Theme 3: Knowing What Is “Normal” and What Is to Be Expected Through Human and Technology Monitoring

A sense of uncertainty was apparent within many patients’ narratives. Uncertainty about symptoms they expected to experience prior to commencing chemotherapy was coupled with uncertainty about what was to be considered “acceptable” or “normal” symptom experiences. For some patients, this uncertainty was tempered by ASyMS because the symptom algorithm “figures that out for you” (United Kingdom, Patient ID 1) and provided patients with clear indications when their symptoms were beyond what was expected:

Because it is really hard to know what you’re supposed to be worried about and what you’re not supposed to be worried about. So, you answer the questions and just kind of say it how it is, then yeah, it kind of figures that out for you. And, you know that if you put anything in then someone will call you...even...I know it did get irritating, but sometimes it is nice to just be able to talk to someone and go is this normal? Do people get this or is this something that needs looking into?

United Kingdom, Patient ID 1

I used it to aid my psyche, when you are not used to being in pain it was helpful to check myself. You get worried, but it gave me information that this might be natural. Getting tired, headaches, feeling unwell. It is a natural part of the treatment. It gave me answers, and I took that in account in my daily life. I did not have to worry about it.

Norway, Patient ID 1

However, for patients who were experiencing symptoms prior to their chemotherapy ASyMS were not sophisticated enough to record these experiences and integrate them into a more personalized alerting algorithm. For some patients, this impacted their interactions with the daily PROMs, and this may be seen to be a limitation of ASyMS.

There was no distinction between symptoms that you experience for the first time and others that you experience as part of your condition. Nowhere did it ask whether you had the symptoms before the treatments or not. But I asked both the doctor and nurse about it. I told them “I had the symptoms from before, so should I write them down?” and they told me “No, do not include them because they continue on without a change.”

Greece, Patient ID 3

#### Theme 4: Enhancing Cancer Care Experiences Through Human and Technology Monitoring

Patients incorporated ASyMS into their daily lives and routines so that it became an integral source of support; many found ways to habituate ASyMS into their routines. It became a companion to them and served as an important facilitator to support their psychological well-being.

It just kind of just became part of my morning routine. I had to get up, have my medication, sit with a glass of water, and do my questionnaire. It didn’t take up a huge amount of time.

United Kingdom, Patient ID 4

And oh my god, for me, when you start to use it, it became part of your life. But, for me it was the safety and that makes me feel so, so good.

Ireland, Patient ID 1599

The enhanced supportive care ASyMS facilitated meant that there was a shift from traditional reactive engagement with clinicians to anticipatory modes of communication. As such, patients across all countries recognized the importance of the alerting algorithms to expedite their care pathways when symptomatic, particularly for out-of-hours care.

Now in fairness, the alert system on it is excellent. I would say I had not put it in five minutes and the phone rang...and it was the hospital and, now it was not that I was waiting for them to ring. But they were that quick, because I would have rang them myself...within the hour but they were back within five minutes...

Ireland, Patient ID 1

I think that this support is absolutely important especially at times when the chemo ward is closed. They do close on a Friday at 4 o'clock and open again on Monday in the morning. There is nothing between that, whom should I call?

Austria, Patient ID 4

#### Theme 5: Informing Future Cancer Care Through Human and Technology Monitoring

A common belief was held by both patients and clinicians that ASyMS has a role in future cancer care delivery with some adjustments and advancements. Some patients suggested that ASyMS is adapted for use beyond acute treatment and is also used for the posttreatment period, particularly for psychological support.

I would like to have it here [follow-up phase] now, too. If not daily, with a different protocol. For example, weekly or when needed. Because the person feels a lot and wishes to know...And sometimes I feel that I am in a worse mood now, when compared how I was feeling during chemotherapy. I do not know if such a boost is available, if there could be psychological support. I would like being able to tell someone how I feel.

Greece, Patient ID 5

However, some patients were frustrated by the static and generic nature of the risk algorithms and so spoke about ways ASyMS could be smarter going forward.

Looking in retrospect, I think that it would have been clever to enter some symptoms so that the statistics is right. I would maybe add that in the programming and make it more sensitive, so that it says that even if you had the symptoms yesterday or last week or have permanently entered them, that one should enter them until. So that the questionnaire could be more explicit about that, and it could be added in there.

Austria, Patient ID 4

Some frustrations with ASyMS were reported by clinicians who believed ASyMS duplicated elements of their current care provision, and others believed specific staffing would need to be in place to facilitate timely responses to ASyMS alerts in the future.

No, it hasn’t changed our [nurse] roles, I can see where it may change roles in centres that don’t have the same structure, we have here within the acute oncology service handling the 24/7 telephone triage. But, for us it hasn’t because we already have a nurse assigned to that and so it is basically just attaching the eSMART role into that existing role.

United Kingdom, Clinician ID 4

I think ASyMS is very important, and I think it worked. It is more on the receiving end to have the appropriate services in place, to have the staff that is going to be responsible for responding to ASyMS. So yes, something like an ASyMS nurse coordinator, or even maybe for the liaison nurses for the different patients, for them to be the alert handlers.

Ireland, Clinician ID 3

## Discussion

### Overview

This study is the first to evaluate, from both patients’ and clinicians’ perspectives, the deployment of a large-scale, international, simultaneous, multicenter, digital remote monitoring intervention in cancer care. Through semistructured interviews and robust qualitative analyses, we identified the positive impact and changes this remote monitoring technology had on patients’ and clinicians’ experiences and clinical practice and areas for improvement for future routine implementations.

### Principal Findings

The qualitative data from patients and clinicians in the eSMART study revealed that both user groups found value in remotely monitoring chemotherapy-related symptoms in real time using the ASyMS intervention. Capturing user experiences across multiple countries and health care systems over a sustained period provides useful insights into the need for, and value of, real-time remote symptom monitoring systems and digitally enabled cancer care models. Patients’ and clinicians’ experiences with ASyMS were largely positive. Most reported value in its purpose and function.

In all the clinical sites, ASyMS was the first implementation of a digitally enabled, patient-driven, nurse-led, remote monitoring model of care. Patients found personal benefits in remotely monitoring their symptoms in real time in their own environments. However, it was the combination of technology and human monitoring that most participants found to be one of the greatest assets of ASyMS. This finding is consistent with the work by Leonardsen et al [19] who interviewed patients with cancer after the rapid implementation of home-based remote monitoring services during the COVID-19 pandemic. In their study, remote monitoring included the use of mobile phones, videoconferences, and daily questionnaire feedback using a tablet or computer. While engagement was positive, it was noted that technologies were used alongside appropriate person-person contact [19]. In a similar way, we demonstrated the value of clinical risk algorithms driving appropriate person-to-person contact through our traffic light triaging system. By alerting clinicians directly, we ensured patients received immediate, effective, tailored, and human symptom management advice.

Compared to previous systems [20-28], the daily reporting, real-time alerting, and integrated feedback components of ASyMS are unique to our remote monitoring system. For example, in a multiclinic RCT evaluation conducted in the United States that evaluated electronic symptom monitoring with patients with cancer [20], patients randomized to the intervention completed patient-reported outcomes once a week through email or automated call prompts. The web-based app eRAPID (Electronic Patient Self-Reporting of Adverse-Events: Patient Information and Advice) used weekly rather than daily symptom reports [21]. The symptom management system [22] filters symptom alerts to members of the research team that can be forwarded to clinicians to respond to within 1-3 days. This delayed response is problematic because of the need for timely clinical interventions. In contrast, our study findings support previous systematic review evidence, which highlights the positive role of daily PROMs in cancer care for patients [23,24]. Such benefits are amplified when the daily PROMs are digitized [23] and when the PROMs facilitate feedback to patients and clinicians [24]. We observed this too in our own study: we mandated response times for amber and red alerts within 8 hours and 30 minutes, respectively, for our digital PROMs, and our qualitative data demonstrate that these parameters and feedback mechanisms were largely acceptable to both patients and clinicians. We do acknowledge, however, that a small number of patients experienced daily PROM completion fatigue and so this may impact on longer-term sustainable adoption. The findings also revealed that some patients and clinicians were frustrated by the static nature of the alerting algorithms. In the future, we plan to use machine learning capabilities to make the algorithms more dynamic, data-responsive, and personalized to adapt to patients’ needs and symptom experiences.

ASyMS was perceived by both patients and clinicians to offer reassurance during chemotherapy largely because of the combined human and technology monitoring afforded by ASyMS. Patients spoke about feeling more knowledgeable about and in control of their symptoms. Clinicians valued real-time monitoring because it did not rely on patients’ retrospective recall. These findings align with previous work that reported positive experiences from both patients and clinicians with the use of real-time remote monitoring systems [20,25-28].

As health care moves toward digitally enabled and digitally driven services, lessons from the successful implementation of remote monitoring systems like ASyMS have increasing importance. In our study, we successfully scaled up and deployed a digital remote monitoring intervention in 5 different countries with over 400 randomized patients. Our interviews with a subsample of patients and clinicians provide us with some important insights for others invested in the challenge of supporting digitally enabled health and care models of the future.

Our system was designed to be accessible to patients and easily integrated into their daily lives to support its use and sustained adoption. Patients’ narratives and reflections highlighted ways in which they had “cognitively habituated” [29] ASyMS into their daily lives and routines. Such habits included keeping the ASyMS device in the same place as a reminder to complete the DCTAQ, identifying a consistent set time each day to complete it, completing it at the same time as taking medications, and setting an alert reminder on another device. These findings support the hypothesis that users of new technologies make changes to their lives to accommodate new digital services in their homes [29]. Therefore, it is important to allow users some flexibility to find their own approaches to “domesticate” the technology to support engagement and sustained adoption.

Shifting health care contexts, particularly following the COVID-19 pandemic, mean that the roles of clinicians are evolving, especially as technology and digital solutions are increasingly integrated into everyday clinical practice [30]. In a recent scoping review [30], the core roles that nurses already have, and will continue to have, in leading digital transformation practices within clinical settings were noted, given their centralized communication role between patients and other clinicians. However, to ensure health care professionals can evolve their competencies within transformative health care contexts, continued professional development opportunities must evolve to allow their effective delivery of meaningful, personalized, and person-centered care, positively enhanced by digital interventions [31]. Our work supports this notion, as we observed noticeable changes in processes of care and roles and responsibilities of clinical staff in countries and clinical settings specifically where a nurse-led, digital health intervention was introduced for the first time.

However, our findings suggest that they would be receptive to more continuous use of remote monitoring technologies in treatment follow-up, given their unmet needs beyond diagnosis and treatment completion [32-34]. Indeed, in our study, some patients identified the period after treatment completion to be the most psychologically challenging because access to care ceases. Thus, a role exists for future digital remote monitoring systems to evolve to support patients during and after cancer treatments. ASyMS is not yet available to monitor long-term cancer experience, but it is a future research priority and concurs with previous identification of patients’ needs for continued use of a weekly symptom reporting system beyond the treatment period [35].

The landscape of health and care service provision is rapidly evolving. An impetus exists to use digital health technologies to influence the ways in which people engage with and receive care. The COVID-19 pandemic necessitated rapid changes in the management of patients with cancer including the increased use of remote patient monitoring technologies to monitor, manage, and engage with patients [36]. However, our work and that of others [37,38] demonstrate that for patients with cancer, a need exists to balance technological remote monitoring interventions and human-human contact to support adoption. Indeed, users of remote monitoring technologies and solutions that were rapidly implemented during the COVID-19 pandemic reported positive perceptions of these technologies, if used in combination with direct person-to-person interactions and support [38]. Therefore, with the unique insights into the experiences of patients and clinicians from 5 very different countries and health care systems that our data afford us, we are confident that technology adoption and acceptability in various health care contexts are most positive when the technology facilitates, rather than replaces, human-human relationships and communications.

### Strengths and Limitations

We interviewed a relatively large sample of patients and clinicians for the qualitative study. Because we interviewed participants in their native language, we avoided a sampling bias of only purposively selecting people who could be interviewed in English. Although successful, this strategy was reliant upon the research teams in each country conducting these interviews. The variability inherent in this approach may have affected the quality and depth of some interviews. However, we mitigated this risk as much as possible by providing thorough preinterview training to personnel, ongoing interview support, and reflective and critical feedback during interviews as part of our quality control measures. We adopted a robust approach to translation and transcription of all interviews to ensure a clear and consistent approach for our data analyses. We are also aware that the patients in this study were predominantly younger age females diagnosed with breast cancer so this may limit the generalizability of our results.

We also acknowledge that our decision to only interview patients in the intervention arm about their symptom management experiences may provide an inflated positive view of the role of daily PROMs and of ASyMS. We did not interview patients in the control group so we do not have comparative narratives, and we did not interview intervention group patients who withdrew from the study. These decisions may mean there is some consequent positivity bias in our findings. However, our large overall qualitative sample from different countries but with similar reported experiences means the validity of our results stands. In addition, attrition in the whole intervention arm in the main RCT was low (34/415, 8.2%, and overall adherence to the intervention was high at 76.9%) [15], providing further support for the largely positive participation experience reported by patients.

### Implications and Conclusions

Successful implementation of digital health interventions can be challenging. We have demonstrated through our qualitative data that patients and clinicians can positively adopt and integrate remote monitoring systems when available to them. Our data demonstrated that ASyMS was, on balance, perceived positively by both patients and clinicians across the eSMART partner countries. Overall, our technology enhanced communications and person-to-person contact between patients and clinicians. The timely symptom management provided by clinicians because of the real-time reporting meant that patients were reassured; gained knowledge about symptoms they were experiencing; and received the right care, at the right time, and in the right place. Going forward, the focus should be on the routine and sustained implementation of scalable, accessible, personalized, and usable digitally enabled cancer care services to help deliver optimal models of cancer care.

## Appendix

Multimedia Appendix 1 Patients' interviews topic guide.

Multimedia Appendix 2 Clinicians' interviews topic guide.

Multimedia Appendix 3 Coding framework.

## Abbreviations

ASyMS: Advanced Symptom Management System
DCTAQ: Daily Chemotherapy Toxicity Assessment Questionnaire
eRAPID: Electronic Patient Self-Reporting of Adverse-Events: Patient Information and Advice
eSMART: Electronic Symptom Management System Remote Technology
PROM: patient-reported outcome measure
RCT: randomized controlled trial

## Data Availability

The datasets generated and analyzed during this study are available from the corresponding author on reasonable request.
