# Supplementary material for: Patients’ and Clinicians’ Experiences Using a Real-Time Remote Monitoring System for Chemotherapy Symptom Management (ASyMS): Qualitative Study
Source: J Med Internet Res. 2024 Dec 3;26:e53834. doi: 10.2196/53834 (PMC11653047; doi:10.2196/53834)
Supplement: Multimedia Appendix 1 [file jmir_v26i1e53834_app1.docx]

***PATIENTS***

Make sure to always probe the participants for more detailed responses. Suggested probes:

- Why?
- What? (examples)
- When?
- Who?
- How?

| **Moderator** | **Question with prompts** | **Probes** |
| --- | --- | --- |
| **Background** | Before we discuss your experiences of the eSMART study in more depth….  Q1. Can you start by tell me why you decided to take part in the eSMART study?  Q2. Can you talk to me about your experiences of using technology before joining the eSMART study?   - Smart phones - Tablets e.g Ipads - Computers | **Because:**  Interested in technology?  Interested in being part of a research study?  The results might help others?  What did your health professional tell you about the study?  Confidence level in use of these? |
| **Perceived Usefulness/**  **Infrastrucutre** | Moving now to your thoughts about the eSMART device.  Q3. What was it like to use the eSMART device?  Please ask about experiences of use across the the different purposes and uses of the device:   - Daily symptom questionnaires - Alerts and responses from clinical teams (red and amber) - The information provided to help you manage your symptoms - Graphs about symptoms - General cancer information and useful contacts - Text messages from health professionals/nurses   Q4. What parts of the eSMART device did you find the **most/least** **useful?** (probe both)   - - Daily symptom questionnaires   - ALERTS?   - The information for managing symptoms   - Graphs about symptoms   - General cancer information and useful contacts   - Text messages from health professionals/nurses   Q5. To help us understand how usable the eSMART device was for you:   - How responsive did you find it to touch? - What was it like to navigate around the device to the different sections (questionnaires, self-care advice, contact information, symptom graphs) - What do you think about the layout of the information on the device?   Q6. Would you make any changes to the eSMART device?   - Daily symptom questionnaires - Alerts and responses from clinical teams (red and amber) - The information provided to help you manage your symptoms - Graphs about symptoms - General cancer information and useful contacts - Text messages from health professionals/nurses   Q7. Would you recommend the eSMART system to other patients with cancer in the future to manage their symptoms?   - - If yes, why? If not, why?   - Would you recommend it to anyone else?   - If you could buy it – would you buy it? Please explain   - Would you have liked it as an app on your own phone? | What? Why? Examples?  Why? How? What? Examples?  How did it make you feel?    Throughout:  What? Why? Examples?  Why? Examples  Change to existing functionality/ new functionalities? Why?  Why? Examples |
| **Other** | Q8: Is there anything you would like to add and we haven’t covered so far about your experiences in the eSMART study? |  |
