# Supplementary material for: Patients’ and Clinicians’ Experiences Using a Real-Time Remote Monitoring System for Chemotherapy Symptom Management (ASyMS): Qualitative Study
Source: J Med Internet Res. 2024 Dec 3;26:e53834. doi: 10.2196/53834 (PMC11653047; doi:10.2196/53834)
Supplement: Multimedia Appendix 2 [file jmir_v26i1e53834_app2.docx]

Appendix 2: Clinicians’ Interviews Topic Guide

***CLINICAL STAFF***

Make sure to always probe the participants for more detailed responses. Suggested probes:

- Why?
- What? (examples)
- When?
- Who?
- How?

| **Moderator** | **Question with prompts** | **Probes** |
| --- | --- | --- |
| **Background** | Before we discuss your experiences of the eSMART study and technology in more depth.  Q1a. Can you talk to me about your role within the eSMART study?   - Nurse - Alert Handler - Other? Please detail   Q1b. What were your previous experiences of using technology to support patients in clinical contexts?   - Smart phones - Tablets e.g ipads - Computers - Online questionaires   Q1c. How would you describe your level of knowledge and experience of using technology generally?   - Learner - Intermediate - Advanced   Q1d. What approaches were used to introduce the eSMART study to your clinical area?   - by whom (role of person, level) - when?   Q1e. What strategies were used throughout the study to keep the study profile in clinician’s minds and maintain their enthusiasm in the study?   - How successful were these approaches? | What previous professional use?  Personal use, /professional use  Training-familiarization  Use of Key Champion / study advocate? |
| **Clinical Experience Changes** | Moving now to focus on your experiences of eSMART:  Q2. What are your thoughts on any ways the eSMART technology makes you work differently in regards to:   - - Symptom management of patients during CTx   - The quality of care deliverd   - Relationships with patients (e.g. time spent with patients)   - Communicating with patients   - Self-management of patients’ symptoms   Q3. In the context of your role, did anything change in your clinical practices for patients who used the eSMART system compared to those who didn’t?   - - Do you do anything now that you didn’t do before?   - Is there anything that you did before that you don’t do now?   Q4. What are your thoughts on the alerting component of the eSMART system?  - Scheduled response time to alerts?  - Risk algorithm and use of red and amber alerts?   - - Were the a) response times and b) risk algorithm practical?   - 24/7 cover   - How long did it take you to respond to alerts?   - Did the alerting component affect your current role positively or negatively?   - Other suggestions for alerting component of the system? | Why?  How?  Better/Worse?  Examples?  Changes in responsibilities? Impact on division of roles?  Change in your role? What?  Probe for examples, why, what, how |
| **Usability** | To help us understand how usable the eSMART system was for you:  Q5a. Thinking first about the nurse handset, what was this like to use from your perspective?    Q5b. Still thinking about the nurse handset, what do you think about:  - mode of delivery (mobile phone)  - visualisation of alerts  - prioritisation and timing of alerts  - the information contained  - the layout  - alerting alarm sound / volume  - like to see any additional features or other  information presented?  Q5c. Thinking now about the eSMART Docobo website, what was this like to navigate?   - - What do you think about:   - the visual appearance  - the information contained  - the layout  - like to see any other information presented on  it?  Q6. Do you have any other suggestions for any changes to the eSMART system that you have not previously mentioned?  - Would you make any modifications to the system you  have not previously discussed?  - Would you add any new functionalities to the system  you have not previously discussed? | Why / Examples  Why / Examples  Why / Examples  What / Why / Examples |
| **Training and Support/Assistance** | Now that we have talked about your experiences of eSMART in the context of clinical care, let’s go back to the training and support you received from the study team….  Q7. Do you think it prepared you sufficently for the use of eSMART?   - - Dealing with system changes/updates   - Key elements of keeping up to date with changes?   - Positive/negative experiences? | Why? What? Examples? |
| **Perceived Usefulness/**  **Infrastrucutre** | Moving now to focus on your perceptions of eSMART.  Q8. What did you:  a) expect prior to using the eSMART technology  b) and what was unexpected when you actually started  using it?  c) What parts of the eSMART system were **most and least**  **useful** for you as a clinician?  [Make sure probe all parts here as these are different questions]  Q9. Have you encountered any:  a) problems, barriers or challenges in using the eSMART  system?  b) enablers, facilitators or solutions to help you use the  eSMART system?   - - If yes, what? What? And how were they resolved? [For both]   - Examples [For both]   Q10. Are there specific parts/functions of the system you are more likely/less likely to use in the future? [Make sure probe both more and less likely]   - - Alerts   - Website   - Self-care advice   Q11. What role do you see for the eSMART technology in clinical practice in the future?   - Would you recommend patients to use it? - Use in other patient groups? - Would you ask/recommend your organisation to buy it for patient care? Please explain - Do you think there is potential for the system to be developed as a smartphone app? | - Why? Examples? - What and Why? Examples - Why? Examples?   What? Why? Examples? |
| **Other** | Q12. Is there anything else about the the eSMART study that is important that we have not discussed? |  |
