# Supplementary material for: Patients’ and Clinicians’ Experiences Using a Real-Time Remote Monitoring System for Chemotherapy Symptom Management (ASyMS): Qualitative Study
Source: J Med Internet Res. 2024 Dec 3;26:e53834. doi: 10.2196/53834 (PMC11653047; doi:10.2196/53834)
Supplement: Multimedia Appendix 3 [file jmir_v26i1e53834_app3.docx]

The coding framework follows from the structure of the topic guides revolving around PARiHS “evidence, context and facilitation” in the implementation of research into practice. Kitson et al. (1998) suggested that implementation of new evidence into practice has as much to do with the context of where the new evidence was being introduced and how that new evidence was introduced, as it has to do with the quality of the evidence. The aim of the interviews was to explore changes in clinical practice and evaluate the short and long-term impact of ASyMS for symptom management in people with the specified cancer types.

The coding framework was organised and reflective of hierarchies in the topic guide such that changes in clinical practice for patients and clinicians was identified, as themes emerged from data collected. It was designed to enable independent data analysis and at the same time, the possibility to compare experiences between patients and clinicians. In addition, given the evolving themes and multiple coders, inter-coder reliability was used to mitigate the potential inconsistencies and disparities in data analysis and interpretation.

A brief description of each main theme is provided below to help coders assess whether data should be coded to that theme/sub-theme or not. In some instances, coded data may also need to include an ‘annotation’ to contextualise a quote to help aid ease of reference across the team. Annotations can be included using Nvivo, and the lead coder can guide people on how to add annotations on Nvivo if required. Table 1 below is a representation of emergent themes and subthemes based on the data collected. This was refined as the analysis progressed and inter-reliability processes were carried out. The analysis is using attributes such as participant demographics and country of the trial.

**Background:** The emergent themes from patients and clinician background for context. This includes the level of confidence and experience with use of technology, and for clinicians particularly, their usual role on the ward. The themes highlight also the prior knowledge and sufficiency/insufficiency of training for the ASyMS technology use.

**Experiences:** The emergent themes from patients and clinician use of the ASyMS technology in clinical practice and care experiences respectively i.e. impact on clinical practice for clinicians and care experiences for patients. The coders defined themes, subthemes and areas around the usefulness for patients care experiences and improvements/impacts and effects on clinical practice for clinicians.

**Recommendations:** The emergent themes on how the ASyMS technology may be improved following from patients and clinicians experience of technology use. The coders used real time experiences of problems with technology use to define the specific themes/subthemes to highlight how the system may be improved for use and full-scale implementation.

## Code-points

| **Title Themes** | **HCP Sub-themes** | **Patients’ Sub-themes** |
| --- | --- | --- |
| Background | **Training**   - Technical Support - Ongoing training - Expectations   **Previous clinical experiences**   - Previous use of technology - Previous digital clinical experience | **Training**   - Technical Support - Ongoing Support - Expectations   **Previous care experiences**   - Previous use of technology - Previous digital care experience |
| ASYMS Experiences | **Pathways of Care**   - Improved Decision making - Provided more details of patients in advance - Impact on clinical care and processes - Time - Targeted care   **Communication**:   - Relationships - Communication pathway - Speedup of contact   **Clinical Risk Algorithm**   - Alerts: too sensitive, confusion, - Timings – odd hours - Response time - Temperature - Blood pressure - Lack of control - Continuous alerts   **Functions**   - Graphs - Tables - Symptoms protocol components - Docobo website   **Usability**   - Sounds - Navigation - Docobo website - User issues & User behaviour - Access to patients data – having to log in   **Challenges**   - Time consuming - Midnight alerts - Patients not picking up phone calls - Technology failure - Carrying two phones | **Quality of Care**   - Reassurance/Security/Safety - Connection to the Hospital - Phone calls - Human Contact - Makes life easier   **Communication**   - Relationship - Contact with staff - Speedup of contact   **DCTAQ**   - Alerts – confusion - Temperature - Blood pressure - Constipation - Diarrhoea   **Functions**   - Self-care advice - Library - Messages - Symptom graphs   **Usability**   - Small text - Charging problems - Complex Navigation - User issues & User behaviour   **Challenges**   - Not enough response options - Time to fill DCTAQ not set - Carrying two phones - Not available for after treatment - Inconsistency - different nurses |
| Recommendations | **Clinical Risk Algorithm**   - Standardise algorithms - Improve alerts   **System/Technology**   - Inbuilt bypass – more control to clinician - Pop up display Screen with details - Intuitive Systems   **Implementation**   - Resources – triage nurses, call centre - Transferability – user targets   **Commercialisation**   - App - Delivery Structure – tiered model, buy/rent | **DCTAQ**   - Set time to fill questionnaire - Add more scale to response options - Improve alerts - Make available for after treatment   **System/Technology**   - Bigger text     **Implementation**   - Resources – triage nurses, call centre - Use beyond treatment - Transferability – user targets   **Commercialisation**   - App - Delivery Structure – tiered model, buy/rent |
